# Thinking in spatial terms: decoupling spatial representation from sensorimotor control in monkey posterior parietal areas 7a and LIP

**DOI:** 10.3389/fnint.2012.00112

**Published:** 2013-01-25

**Authors:** Matthew V. Chafee, David A. Crowe

**Affiliations:** ^1^Department of Neuroscience, University of Minnesota Medical SchoolMinneapolis, MN, USA; ^2^Brain Sciences Center, VA Medical CenterMinneapolis, MN, USA; ^3^Center for Cognitive Sciences, University of MinnesotaMinneapolis, MN, USA; ^4^Biology Department, Augsburg CollegeMinneapolis, MN, USA

**Keywords:** spatial cognition, spatial attention, area 7a, LIP, parietal cortex, object-centered, constructional apraxia, navigation

## Abstract

Perhaps the simplest and most complete description of the cerebral cortex is that it is a sensorimotor controller whose primary purpose is to represent stimuli and movements, and adaptively control the mapping between them. However, in order to think, the cerebral cortex has to generate patterns of neuronal activity that encode abstract, generalized information independently of ongoing sensorimotor events. A critical question confronting cognitive systems neuroscience at present therefore is how neural signals encoding abstract information emerge within the sensorimotor control networks of the brain. In this review, we approach that question in the context of the neural representation of space in posterior parietal cortex of non-human primates. We describe evidence indicating that parietal cortex generates a hierarchy of spatial representations with three basic levels: including (1) sensorimotor signals that are tightly coupled to stimuli or movements, (2) sensorimotor signals modified in strength or timing to mediate cognition (examples include attention, working memory, and decision-processing), as well as (3) signals that encode frankly abstract spatial information (such as spatial relationships or categories) generalizing across a wide diversity of specific stimulus conditions. Here we summarize the evidence for this hierarchy, and consider data showing that signals at higher levels derive from signals at lower levels. That in turn could help characterize neural mechanisms that derive a capacity for abstraction from sensorimotor experience.

## Introduction

Human cognition, or in colloquial terms, thinking, is notoriously difficult to define. However defined, thinking has to be a property of neurons, and it might be possible to infer several basic and simple features of the neural mechanisms responsible without a final or complete description of the cognitive processes themselves. For example, it seems impossible to provide any biological account for thinking without patterns of activity in the cerebral cortex exhibiting the property of sensorimotor independence. That is, in order to think, the brain must be able to internally generate a sequence of patterns of neuronal activity that encode behaviorally useful information independently of concurrent sensory or motor processing. Sensorimotor independence, as we will refer to this property, seems a necessary starting point (without which thoughts would be confined to the set of current stimuli and actions). Second, it seems reasonable to assert that in many primates (including humans) some forms of thinking, associated with the intelligent control of behavior, involve abstraction (Miller, 2000; Tenenbaum et al., 2011). More specifically, the brain has to be able to generate patterns of neuronal activity that encode a particular type or class of information, best characterized by the property of generalizability. Neural signals engaged in abstraction encode regularities, relationships, or principles of general applicability that apply to a wide variety of particular sensory or motor conditions, and that capability at the single neuron level is likely to enable the brain to predict outcomes based on principles applied in novel circumstances, one of the defining central characteristics of intelligence.

The purpose of this review is to evaluate what we know about abstraction and sensorimotor independence specifically as it applies to the internal representation of space by neurons in the posterior parietal cortex of non-human primates. Available experimental evidence obtained from single neuron recording studies in parietal cortex has documented a rich diversity of spatial representations that should facilitate investigation into the neural mechanisms by which abstraction and sensorimotor independence are built on top of, or derive from, more basic sensorimotor signals in the brain. For example, a long experimental history has established the strong relationship between neural activity in the parietal cortex and the representation of spatial information that either derives from sensory input or that predicts forthcoming movement. The relative importance of visual processing and motor processing in parietal cortex has been debated for more than 30 years (see below), but there seems some consensus that parietal cortex is interposed between sensory input and motor output and is likely to play a role converting sensory representations into motor representations. Second, a number of recent studies have shown that under certain circumstances, the same neural architecture that mediates spatial sensorimotor control is also capable of mediating spatial cognition. These data show that when confronted by more complex spatial problems, (such as those which require analyzing spatial relationships or computing spatial categories, for example), parietal neurons exhibit new forms of spatial representation more closely related to spatial reasoning or spatial problem solving than spatial sensorimotor control. Because spatial representations within parietal cortex span the range from concrete sensorimotor to abstract cognitive, parietal cortex offers a unique opportunity to gain insight into one of the most basic questions in cognitive neuroscience: how neural systems that perform a specific role in sensorimotor control acquire the capacity for abstraction and sensorimotor independence. The answer to that question is likely to lead to a greater understanding of how human intelligence emerged within a sensorimotor architecture such as the cerebral cortex.

The review is divided into four sections. The purpose of the first three sections is to review the evidence that three distinct types of neuronal signals coding spatial information coexist within posterior parietal cortex that can be considered to constitute the levels of a hierarchy of spatial representation. (The hierarchy is defined by the nature of the spatial information encoded at each level rather than then the neuronal populations engaged, to acknowledge that single neurons can carry a mixture of signals and participate at multiple levels of representation.) At the first level (which we refer to as first order spatial coding), neural signals encode stimulus attributes and movement parameters, and spatial processing faithfully reflects ongoing sensorimotor control. This is exemplified by the familiar spatial tuning of single neuron activity for stimulus position or movement direction, and the population representation of these parameters (Mountcastle et al., 1975, 1981; Georgopoulos et al., 1982, 1988; Andersen et al., 1985; Schwartz et al., 1988), which together probably represent the most behaviorally crucial forms of spatial representation in the brain. At the second level (second order), the spatial information coded by neural activity retains its dependence on stimulus attributes (such as position) and movement parameters (such as direction), so from a spatial perspective, activity does not exhibit sensorimotor independence. However the duration and intensity of these signals are modulated as a function of cognitive factors. Working memory, attention, motor planning, and decision-processing can all be characterized as instances of second order spatial processes on that basis. At the top level, neural activity encodes spatial information that exhibits complete sensorimotor independence, in both temporal and spatial domains. At this level, neurons carry signals that convey abstract, generalized spatial information, such as spatial relationships or spatial categories that generalize across numerous stimulus configurations, and no longer pertain to specific stimuli or movements. After considering the evidence that all three types of spatial representation coexist in posterior parietal cortex, we will address (see section “Origin of third order spatial representations”) how abstract spatial information encoded at upper levels of this hierarchy might derive from transformations applied to spatial information present at lower levels, and speculate as to what the neural mechanisms that mediate interactions between these levels of processing might be. The issue of how signals that reflect more abstract forms of cognition emerge in sensorimotor control networks (such as the posterior parietal cortex or the cortex in general), perhaps as a consequence of sensorimotor experience, is an important question, though relatively little is presently known in terms of underlying neural mechanisms. We hope that dissociating stages and types of spatial codes that exist within parietal cortex may facilitate discovering more about how they are generated by an interaction between the neural systems of the cerebral cortex and a spatially structured environment.

### First order spatial coding: sensorimotor control in posterior parietal areas 7a and LIP

We focus the review on experimental data obtained in two adjacent parietal subdivisions, area 7a, which is located in the posterior part of the inferior parietal lobule, and area LIP, in the lateral bank of the intraparietal sulcus. A number of studies investigating the neural mechanisms of spatial cognition in parietal cortex in monkeys have focused on these two areas, providing a good basis for a comparison between cognitive and sensorimotor information processing within them.

#### Area 7a

Area 7a is located in the posterior aspect of the inferior parietal gyrus in monkeys. Recent work in this area has focused on its role in various forms of spatial cognition (attention, working memory, and more abstract processes), but its direct involvement in basic visual processing, specifically spatial visual processing, has been firmly established by prior research. (The role of area 7a in motor processing is less well-understood). For the purpose of establishing the coexistence of sensory and cognitive signals coding spatial information in this area, we briefly review some of the evidence indicating involvement of area 7a in first order spatial coding in the visual modality. The defining characteristic of the visual sensory responsiveness of area 7a neurons is that neuronal activity is tuned primarily with respect to the spatial attributes of visual stimuli—where they are located on the retina and how they are moving. Area 7a neurons can often be robustly driven by visual stimuli independently of cognitive factors. The visual receptive fields of 7a neurons are large and in many cases bilateral (Blatt et al., 1990), and can be driven either by stationary visual stimuli (Yin and Mountcastle, 1977; Robinson et al., 1978; Motter and Mountcastle, 1981; Mountcastle et al., 1981; Constantinidis and Steinmetz, 2001a, 2005) or moving visual stimuli (Motter et al., 1987; Steinmetz et al., 1987; Merchant et al., 2001, 2003, 2004a,b; Raffi and Siegel, 2007). Motion sensitive receptive fields of area 7a neurons often exhibit a radial arrangement of preferred directions throughout their receptive field (Motter and Mountcastle, 1981; Steinmetz et al., 1987), such that these neurons are maximally activated by either expanding or contracting patterns of optic flow (Siegel and Read, 1997; Merchant et al., 2001, 2003; Raffi and Siegel, 2007), as occurs when the observer moves through a fixed visual environment. It has been recently noted that visual motion information in parietal area 7a could be used to derive the positions of visual landmarks and the location of the observer with respect to those landmarks, a type of spatial processing important for navigation and spatial orientation (Kravitz et al., 2011). Collectively these data indicate that area 7a neurons carry a rich array of physiological signals encoding spatial attributes of visual stimuli even under conditions (in many cases) where those stimuli are passively presented and do not have a direct behavioral or cognitive significance. As discussed in subsequent sections these signals are frequently modulated by cognitive factors, but cognitive processing *per se* is not a necessary precondition for the activation of area 7a neurons by visual stimuli.

Visual neurons in area 7a exhibit another characteristic that provides substantial insight into the spatial functions of parietal cortex in general. Many area 7a neurons exhibit gain fields (Andersen and Mountcastle, 1983; Andersen et al., 1985, 1990b), the term given to describe the influence of eye position on visual sensitivity. These neurons possess visual receptive fields that remain fixed in position in relation to the fovea, but the sensitivity of the receptive field is a systematic function of the position of the eyes in the orbits at the time that the visual stimulus is delivered (Andersen and Mountcastle, 1983; Andersen et al., 1985, 1990b). This provides an example of parietal neurons integrating diverse types of sensory information to construct superordinate spatial representations—body-centered spatial representations in this case (Andersen, 1997)—which can then be used to direct movement (Andersen and Buneo, 2002; Buneo and Andersen, 2006). The visual sensitivity of area 7a neurons is modulated not just by eye position but by other postural factors, such as the position of the head with respect to the environment, a type of spatial tuning that could help to construct a “world-centered” representation of space (Snyder et al., 1998b). Spatial representations of this class, specifying the location of visual targets relative to the body, or the world, constructed by integrating information that derives from the retina as well as a variety of somatosensory sources, has direct utility for the visual control of movement. From this perspective then, posterior parietal cortex is a prototypical sensorimotor cortex.

#### Area LIP

**Figure 1:**
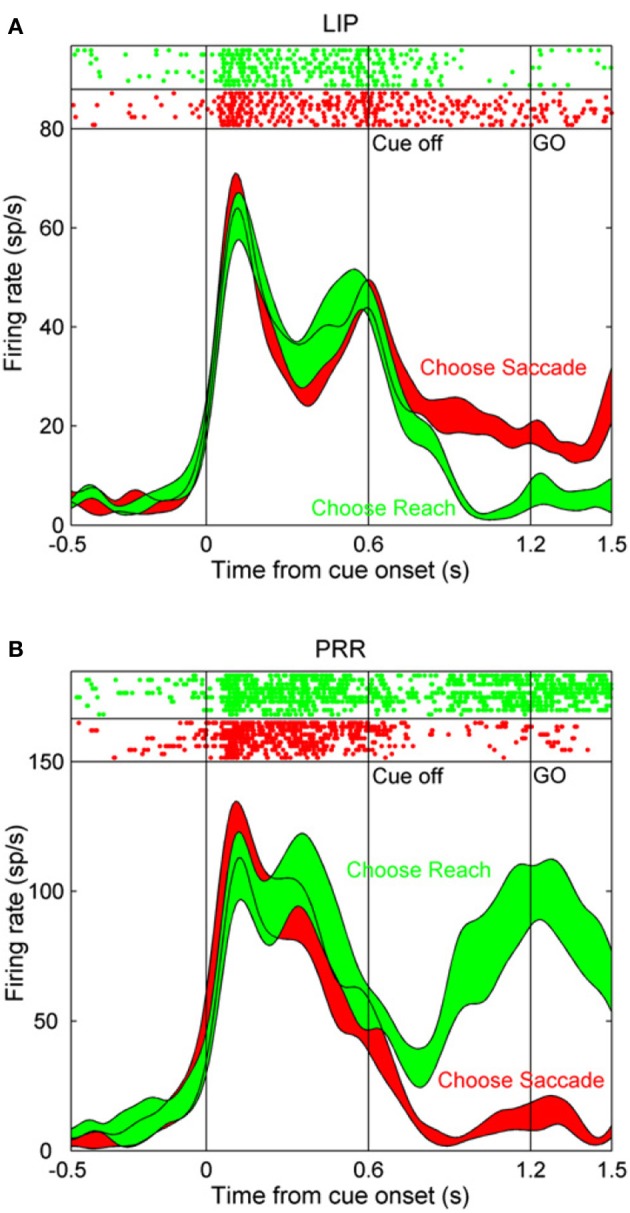
**The activity of single neurons in the lateral intraparietal (LIP) area and in the parietal reach region (PRR) during a task in which monkeys autonomously decided to make a saccade or a reach toward the same remembered visual target location. (A)** After the initial visual transient at the onset of the peripheral cue, activity in this LIP neuron is greater during the following delay period on trials that the monkey decided to make an eye movement (red trace) than an arm movement (green trace). **(B)** Corresponding data in a PRR neuron. Activity in this case was greater on trials that the monkey decided to make an arm movement relative to a saccade. Reprinted with permission (Cui and Andersen, 2007).

The lateral intraparietal area (LIP) is located just medial to area 7a, in the lateral bank of the intraparietal sulcus. This area was first identified on the basis of its particularly strong anatomical connection with the frontal eye fields and the presence of neurons with presaccadic activity (Andersen et al., 1990a). Subsequent neural recording experiments have confirmed a role in saccade control. Many LIP neurons are activated before the initiation of saccades and their firing rate varies systematically as a function of saccade direction (Barash et al., 1991). In the case that the saccade is delayed for several seconds after the disappearance of a visual target, LIP neurons maintain spatially selective activity for the intervening working memory period until the saccade is executed (Gnadt and Andersen, 1988; Chafee and Goldman-Rakic, 1998). Although it can be problematic to dissociate visually evoked activity from motor-related activity when movements are made toward visual targets, (or to dissociate motor plans from spatial attention under these circumstances), the activity of many LIP neurons maintains a relationship to the direction of the forthcoming saccade in double-step tasks in the case that no visual stimulus appeared in the movement field of the neuron (Mazzoni et al., 1996). This demonstrates that a visual stimulus is not necessary for LIP neurons to exhibit spatially selective activity, as is further indicated by the finding that LIP neurons are active before memory-guided saccades to auditory stimuli (Stricanne et al., 1996). Moreover, the activity of LIP neurons is frequently effector specific, and is greater when monkeys plan and execute saccadic eye movements than when they make reaching arm movements to the same visual targets (Snyder et al., 1998a; Quian Quiroga et al., 2006). Neurons in the parietal reach region (PRR) in the medial bank of the intraparietal sulcus are likewise effector specific, but are more strongly activated before arm movements than saccades. Interestingly, neural activity in these two structures is modulated as a function of which effector monkeys autonomously decided to move to a cued spatial location under conditions in which they randomized their choice of effector (Cui and Andersen, 2007). Figure 1, taken from (Cui and Andersen, 2007), illustrates this phenomenon. Following the initial visual transient, which was of comparable magnitude regardless of the effector selected, activity in the LIP neuron was higher on trials that the monkey decided to make a saccade to the remembered target location (Figure 1A; red trace), in comparison to when it decided to make a reach to the same location (Figure 1A; green trace). Activity in the PRR neuron exhibited the converse pattern, and was more strongly active on trials that the monkey decided to make an arm movement (Figure 1B; green trace) rather than a saccade (Figure 1B; red trace). Because visual stimulation and attention are likely to be comparable whether a monkey executes a saccade or a reach to the same visual target, effector specificity argues that visual input and attention alone cannot entirely account for the activity of LIP (and PRR) neurons.

A selective relation between LIP activity and saccades is further documented by the finding that these neurons are more strongly driven by central cues that instruct saccades vs. reaching arm movements, even in the case that the direction of the eye movement is not known prior to the appearance of the central cue (Dickinson et al., 2003). Spatial coding of saccade direction in LIP is also modulated by eye and head position, suggesting that LIP contributes to body-centered representations of space (Snyder et al., 1998b), and there is evidence that this spatial representation is three dimensional (Gnadt and Mays, 1995) and is topographically organized (Blatt et al., 1990; Patel et al., 2010; Savaki et al., 2010). Finally, it is possible to trigger saccades by electrical microstimulation of area LIP (Thier and Andersen, 1998), though higher currents are typically required in comparison to the frontal eye fields. A role for LIP in saccade control is generally consistent with its connectional anatomy. LIP output projections target saccade-related structures such as the frontal eye fields (Cavada and Goldman-Rakic, 1989b; Blatt et al., 1990) and the superior colliculus (Pare and Wurtz, 2001). The nature of that role—whether to select visual targets or spatial locations to guide downstream oculomotor structures or to provide an explicit motor command itself—though, remains a point of controversy. However, if an area codes spatial information that is dedicated to a particular motor output pathway (such as one that controls saccadic eye movements), as the effector specificity of LIP activity appears to suggest, then the distinction between these alternatives becomes difficult to precisely define.

Although the above evidence indicates that neural activity in area LIP relates to the direction of upcoming saccades, by the same token, a substantial body of evidence indicates that saccade control by itself cannot entirely explain the neural representation of space in this area. Neurons in area LIP exhibit short-latency ON responses that are tightly coupled to the appearance of visual stimuli (Bisley et al., 2004), and respond to visual stimuli even in the case that they do not serve as saccade targets (Colby et al., 1996; Platt and Glimcher, 1997; Powell and Goldberg, 2000; Gottlieb et al., 2005; Premereur et al., 2011). By comparing activity of LIP neurons when saccades are made toward and away from visual targets (anti-saccades), it is possible to determine whether the spatial selectivity of neurons is related to the position of the visual stimulus or the direction of the forthcoming eye movement. Under these circumstances, the activity of most LIP neurons reflects the position of the visual stimulus serving as the saccade target and not the direction of the pending saccade (Gottlieb and Goldberg, 1999), although there is some evidence that an initially stimulus-bound spatial signal in LIP converts to a more closely saccade-bound signal as the delay period progresses, and the time of the pending saccade approaches (Zhang and Barash, 2004; Gottlieb et al., 2005). All of these observations indicate the presence of a visual representation in area LIP that does not bear an obligatory relation to the direction of saccades. In fact, LIP neurons can show selectivity for the shape of visual stimuli (Sereno and Maunsell, 1998; Lehky and Sereno, 2007; Janssen et al., 2008), a type of visual feature selectivity that shows a role for LIP in visual processing that extends beyond saccade control.

Although the precise balance of sensory and motor processing in area LIP (and in parietal cortex in general) remains to be determined, there seems little doubt, given that both factors influence activity in posterior parietal cortex, that this area is intrinsically sensorimotor cortex and, as a result, its function is not entirely reducible to one side of this continuum (sensory or motor) considered in isolation of the other. Additional evidence (reviewed below), argues that these same parietal areas are able to also participate in cognitive processes that to various degrees are abstracted from sensorimotor control.

### Second order spatial coding: cognition as modulation of the timing and strength of sensorimotor signals

A brain confined to processing current sensory input and motor output would be of limited intelligence. Human mental activity, and its contribution to intelligent behavior, depends directly on the capability of cortical systems to represent and process information that is decoupled from sensorimotor control, both in time and in information content. In this section, we consider how relatively simple modifications of sensory and motor signals in posterior parietal cortex can implement a diverse set of sophisticated cognitive processes, including spatial attention, spatial working memory, and decision-processing. The neural correlates of each of these cognitive processes can be understood to emerge by a modification of either the strength or timing of sensory and motor signals in the brain. In each case however, the spatial information coded by neural activity remains tightly coupled to specific stimuli or movements.

#### Spatial working memory

The spatial delayed response task, which requires monkeys to direct a motor response toward a cue or stimulus that was seen in the recent past (but is not visible at the time of the motor response) is a classical test of spatial working memory in monkeys (Goldman-Rakic, 1988, 1995). An oculomotor variant of this task (Funahashi et al., 1989), the memory-guided saccade task, requires monkeys to make memory-guided saccades toward the location of a brief visual target several seconds after it has disappeared. During the performance of memory-guided saccades, neurons in parietal area LIP are tonically activated for the interval of time between the presentation of the visual stimulus and the subsequent delayed saccade (Gnadt and Andersen, 1988; Chafee and Goldman-Rakic, 1998). This neural activity appears to play a role in spatial working memory, in the sense that it spans the delay period between stimulus and response and is selective for the spatial information needed to direct that response. Other groups have shown that area 7a contributes to sensory-based spatial working memory (Constantinidis and Steinmetz, 1996; Qi et al., 2010; Rawley and Constantinidis, 2010). Delay activity in parietal cortex observed on spatial working memory remains tightly coupled to stimulus position or movement direction (identifying it as a correlate of a first order spatial cognitive process by the definition above). Only the timing of neural activation with respect to external sensorimotor events has changed.

#### Spatial attention

Much of the history of posterior parietal research over the last 35 years has been defined by the intention-attention debate, the question as to whether the primary function of this cortical area is to formulate motor plans (Mountcastle et al., 1975; Snyder et al., 1998a, 2000; Quian Quiroga et al., 2006) or to allocate spatial attention (Robinson et al., 1978; Bushnell et al., 1981; Gottlieb et al., 1998; Bisley and Goldberg, 2003, 2006). The two alternatives have proven to be extremely difficult to dissociate experimentally. One reason is that the motor function of parietal neurons in monkeys has often been studied by having monkeys make movements toward visual targets, which suddenly appear at unpredictable locations, and as such are likely to draw bottom-up attention to the stimulus. In addition, spatial attention and motor planning may be functionally linked (Hoffman and Subramaniam, 1995; Deubel and Schneider, 1996), a view articulated by the premotor theory of attention (Rizzolatti et al., 1987). A role for parietal cortex in spatial attention is clearly indicated by the observation that patients with parietal lesions exhibit spatial neglect, a condition in which they fail to consciously perceive stimuli delivered contralateral to their damaged cortical hemisphere (Husain and Nachev, 2007; Corbetta and Shulman, 2011).

Recordings in area 7a have provided evidence that neural activity in this area generates signals that specify where attention should move. The visual responses of 7a neurons are suppressed if attention is already located at the cells' visual receptive field when the stimulus appears, but are robust if attention is directed elsewhere, a finding which could indicate that 7a neurons are activated when the location of attention is shifted (Steinmetz et al., 1994; Robinson et al., 1995; Constantinidis and Steinmetz, 2001b). A similar mechanism could account for the observation that 7a neurons are activated to encode the location of salient stimuli that pop-out form other stimuli in a visual array by virtue of being visually distinct, and therefore drawing attention (Constantinidis and Steinmetz, 2001a, 2005). However, the relation between attention and neural activity in area 7a is complex, and dependent on training. For example, in monkeys trained to base their responses on the position of a stimulus defined in an external frame of reference (rather than the retinal location of the stimulus), neural responses at attended locations are enhanced rather than suppressed (Rawley and Constantinidis, 2010). These data indicate that the relation of neural activity to attention in area 7a is plastic and could reflect the spatial coordinate system the brain has been trained to employ (Chafee et al., 2007), however the nature of task effects on attention-related activity in area 7a is not yet fully understood.

**Figure 2:**
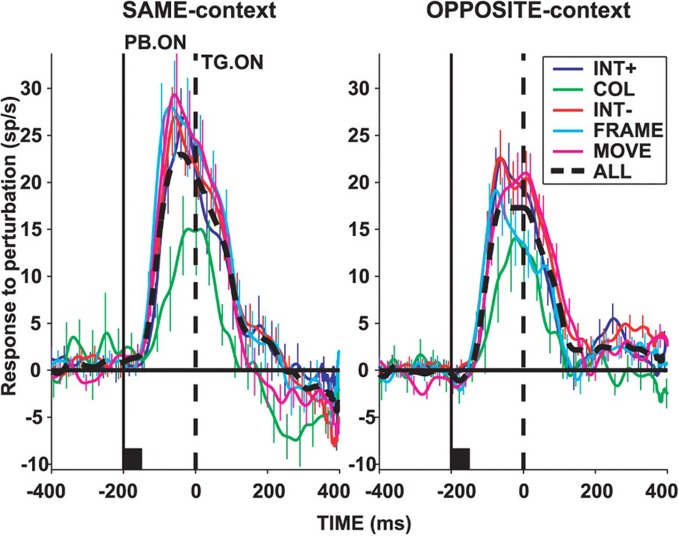
**Population activity in area LIP of monkeys performing a covert visual search task using a stable stimulus array.** Activity functions plot the difference in mean LIP neuronal population firing rate when the stimulus in the receptive field (RF) underwent a salient perturbation vs. when it did not. Upward deflections indicate an increase in firing rate caused by the salient visual event regardless of whether the search target was located inside (“SAME-context”), or outside (“OPPOSITE-context”) the receptive field. Visual perturbations included an increase (“INT+”), or decrease (“INT−“) in stimulus intensity, a change in color (“COL”), a shift in stimulus position (“MOVE”), or appearance of a bounding frame (“FRAME”). In each case, the visual perturbation was task-irrelevant and had no bearing on response selection. Reprinted with permission (Balan and Gottlieb, 2006).

In area LIP, neurons are activated by visual stimuli that appear abruptly in their visual receptive fields even in the case that monkeys never make a saccade toward the stimulus (Gottlieb et al., 1998; Kusunoki et al., 2000). Moreover, it appears that the abrupt onset of the stimulus, and the potential capture of bottom-up attention, accounts for a large part of the neural response, as LIP neurons do not respond to the presence of identical stimuli brought into their receptive fields by a saccade (Gottlieb et al., 1998; Kusunoki et al., 2000). The activity of LIP neurons is reduced before saccades made without a visual target, and is augmented if the visual stimulus is their receptive field is made task-relevant (Gottlieb et al., 1998; Kusunoki et al., 2000). Subsequent studies have shown that LIP neurons respond briskly to visual events in their receptive fields that grab attention but have no other behavioral significance in terms of instructing a required motor response (Balan and Gottlieb, 2006). Figure 2, taken from (Balan and Gottlieb, 2006), illustrates this effect in LIP neurons studied during a covert visual search task. Population activity functions plot the increase in firing rate of LIP neurons when the stimulus in their visual receptive was briefly perturbed (for example by shifting color or changing position slightly). Each of these visual events had no bearing on the type or direction of the required motor response, yet each produced an increase in the activity of LIP neurons. These data provide evidence that LIP neurons can be driven by visually salient stimuli, regardless of their motor significance. Conversely, the responses of LIP neurons to visual stimuli are suppressed if those stimuli are overtly ignored (Ipata et al., 2006). These and other data support the view that area LIP generates a salience map of visual space (Goldberg et al., 2006; Gottlieb, 2007).

To directly examine whether neural activity in LIP observed during motor planning tasks may reflect the location of spatial attention, Bisley and Goldberg presented a probe stimulus in the middle of a memory-guided saccade trial, finding that attention was located at the position of the saccade target, which was the location coded by the concurrently active population of LIP neurons (Bisley and Goldberg, 2003). These authors also found that the tight correspondence between the location of spatial attention and the location coded by neural activity in area LIP persisted when attention was transiently drawn to a distractor stimulus, even though this never served as the target for a movement (Bisley and Goldberg, 2003).

Collectively, these data provide strong evidence that neural activity in area LIP has a role in visual attention that can, with experimental care, be dissociated from motor planning. However, the data do not seem to preclude that neural activity in LIP provides spatial targeting information preferentially to the oculomotor system (via output projections to the frontal eye fields and superior colliculus, for example). It seems likely, given the quantity of evidence on both sides of the debate, that attention and intention colocalize to posterior parietal cortex, and may represent two sides of one coin, in the sense that a spatial bias signal originating in parietal cortex could simultaneously influence processing in motor and sensory areas that receive parietal input (Cavada and Goldman-Rakic, 1989a,b; Andersen et al., 1990a; Wise et al., 1997; Marconi et al., 2001; Tanne-Gariepy et al., 2002) albeit to different degrees depending on task conditions. If the fundamental role of parietal cortex is to derive spatial information from the sensory input and relay this spatial information to motor systems, it would seem advantageous if the spatial representation were selective, restricted to the most salient or behaviorally relevant stimuli, to prevent motor systems from being inundated with more spatial targeting information than they could effectively translate into movement at any given instant.

In all of the above studies, regardless of whether the neural activity observed reflected a motor plan, a map of behavioral salience, or a shift in covert attention, the spatial information coded by that activity related directly to the position of a visual stimulus or the direction of a forthcoming movement. In this regard, spatial attention qualifies as a second order spatial process by the definition above. The neural representation is a joint function of sensorimotor and cognitive factors, but the spatial content of the neural representation maintains a close relationship to stimulus position or movement direction. In these instances, then, spatial cognition rests upon a neural mechanism that is only partially decoupled from sensory processing or sensorimotor control. The neural correlate of spatial attention in this case consists essentially of a variable gain imposed by a cognitive process on a fundamentally sensory signal.

#### Spatial decision-processing

Neural recordings in posterior parietal cortex of monkeys during decision-making tasks have provided crucial insight into the neural mechanisms involved, and in most of these studies, the neural mechanisms of decision-processing have reflected a second order spatial process as defined above. In one widely used paradigm, monkeys make a decision to saccade in a particular direction based the predominant direction of visual motion in a field of moving dots. By systematically varying the proportion of dots moving in the same direction, it is possible to produce motion percepts of graded strength (Newsome et al., 1989). Under these conditions, LIP neurons are more strongly active before saccades in their preferred direction if the decision is based on a stronger motion percept (Shadlen and Newsome, 1996, 2001; Roitman and Shadlen, 2002; Churchland et al., 2008; Kiani and Shadlen, 2009). This provides evidence that LIP activity scales with the confidence or certainty of a spatial decision. Under a control condition in which the dots move in random directions (and there is no coherent motion percept) monkeys saccade in variable directions. The fact that LIP activity continues to predict saccade direction in this case (Shadlen and Newsome, 1996) makes it difficult to interpret the activity as reflecting visual salience or attention only, as neural activity predicts the variable saccade direction over trials in which the positions of visual targets and the features of the motion stimulus do not vary. Subsequent studies have refined our understanding of the neural mechanisms that mediate the decision, providing evidence that LIP neurons integrate motion information over time (Huk and Shadlen, 2005), and that once activity in LIP reaches a boundary, the saccade is executed (Kiani et al., 2008). In the most widely used version of the moving dot perceptual decision task, the perceived direction of visual motion (the perceptual decision), and the direction of the saccade (the motor decision) are coupled, making it difficult to determine whether neural activity reflected spatial aspects either of the stimulus or the required motor response. In a recent study dissociating these two spatial variables, visual motion and saccade planning directions independently modulated the activity of single LIP neurons (Bennur and Gold, 2011), confirming a role for parietal cortex in both visual and motor processing.

**Figure 3:**
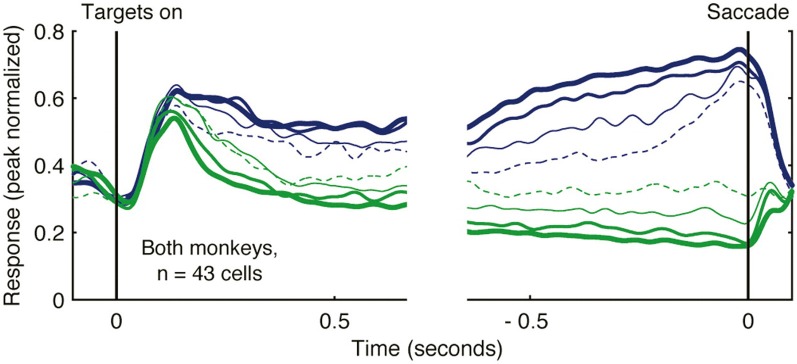
**Activity of LIP neurons scales with action value in a decision-making task.** Monkeys decided whether to saccade toward one of two alternative targets (red or green) under conditions in which the reward that each target would deliver changed dynamically as a function of choice and reward history. Spike density functions plot the mean firing rate of 43 LIP neurons as a function of the local fractional income, or the proportion of recently earned reward attributed to the color of the saccade target, that the monkey selected. Activity when the monkey decided to saccade toward the target in the receptive field is illustrated in blue, and to the target away from the receptive field in green. Lines of increasing thickness indicate greater fractional income associated with the selected target. Reprinted with permission (Sugrue et al., 2004).

Rather than varying the strength of sensory evidence, other studies of decision-making have systematically varied the magnitude or probability of reward. This approach has successfully demonstrated that increasing reward magnitude or probability enhances the strength of saccade planning activity in LIP (Platt and Glimcher, 1999). Subsequent studies simultaneously manipulating both the strength of sensory evidence and the magnitude of reward have shown that both factors influence motor planning activity in LIP (Rorie et al., 2010). Under real world conditions, decisions are often not dictated by explicit sensory cues, but rather reflect varying estimates of action value based on past decisions and outcomes. Under these conditions, neural activity in LIP reflects a temporally local (and continuously varying) estimate of action value (Sugrue et al., 2004). Neural activity showing this relationship is illustrated in Figure 3. In this experiment, the authors derived an estimate of the subjective value that monkeys assigned to alternative actions (the local fractional income) which reflected how much reward monkeys had earned for a given action in the recent past, and that accurately predicted their subsequent choices. Activity functions in Figure 3 illustrate the firing rate of a population of LIP neurons when their preferred saccade was associated with different values. As the local fractional income of the saccade target increased, the intensity of LIP activity increased also (Figure 3; blue activity functions of increasing thickness). These data provide clear evidence that neural activity in LIP reflects not only saccade direction but also the value attributed to the saccade.

Other studies have shown that LIP neurons are involved in aspects of decision-processing that extend beyond the evaluation and neural representation of action value. For example, in monkeys adjusting their response strategy to beat a computer opponent in a free-choice oculomotor game, LIP neurons encode both the current value of alternative actions, as well as actions and outcomes on prior trials, information that could play a role in adjusting strategy to counteract the computer opponent (Seo et al., 2009). Finally, the activity of LIP neurons bears a basic relation to reward prediction, even when the reward is not a consequence of a particular action. For example, neurons in this area emit stronger responses to visual stimuli that signal the delivery of reward relative to stimuli that do not, even when the location of the stimulus does not bear any relation to the direction of the saccadic response (Peck et al., 2009).

Scaling motor plans as a function of value or anticipated reward could be expected to bias the competition among alternative motor plans in favor of the action with the highest payoff. This formulation of decision-processing bears a strong resemblance to the biased competition model of visual attention, in which attention biases the competition between multiple stimulus representations in favor of those which are most salient or behaviorally relevant (Desimone and Duncan, 1995). The finding that expected reward can also modulate visual signals in area LIP (Peck et al., 2009), in addition to motor signals as indicated by the above studies of decision-processing, suggests that visual attention and decision-processing may be mediated by similar neural mechanisms (Gottlieb and Balan, 2010).

From the perspective of spatial sensorimotor independence, the above studies characterize decision-processing as a second order spatial process. The data show that LIP activity that codes the direction of the next saccade is modulated in strength according the predicted outcome or subjective value of an action. However, the influence of reward or value-related cognitive variables on neural activity does not force the spatial representation itself in LIP away from a tight relationship to the spatial aspects of sensory input or motor output. More specifically, the spatial information coded by neural activity in the majority of these studies continues to represent the spatial features of particular visual stimuli (e.g., the position or direction of motion of visual stimuli), or the spatial features of particular movements (e.g., the direction of a planned saccade).

### Third order spatial processing: decoupling spatial representation from sensorimotor control

As indicated by the experimental findings reviewed above, a rich variety of spatial cognitive operations can be achieved by modulating the duration or intensity of neural signals that code stimulus position or movement direction. In this section we will consider the evidence that neural representations of space in posterior parietal cortex can be decoupled from sensory and motor processing to support more abstract forms of spatial cognition. Our interest is to understand how spatial information which is abstracted from sensory or motor processing is represented by the activity of parietal neurons and is utilized to direct spatially intelligent behavior. A rapidly growing body of evidence indicates that posterior parietal neurons participate in a broad range of functions that extends beyond the boundaries of spatial attention or sensorimotor control, to provide neural representations of abstract cognitive variables such as numbers (Nieder and Miller, 2004), rules (Stoet and Snyder, 2004), categories (Freedman and Assad, 2006; Goodwin et al., 2012; Swaminathan and Freedman, 2012), and time (Leon and Shadlen, 2003; Janssen and Shadlen, 2005). Here we will focus on studies providing evidence that the computational capacity of parietal neurons extends to include abstraction in the spatial domain, characterized by neural signals that code spatial information related to the solution of spatial cognitive problems rather than spatial sensorimotor control.

#### Spatial representation during route traversal

One spatial cognitive task that generates abstract spatial representations in parietal cortex is the traversal of routes. Recent human imaging studies have found that parietal cortex is activated when subjects must navigate through an environment (Shelton and Gabrieli, 2002; Rosenbaum et al., 2004; Wolbers et al., 2004; Spiers and Maguire, 2007; Ciaramelli et al., 2010). This activity is often characterized as reflecting spatial processing in egocentric coordinates. In non-human animals, however, there is evidence to indicate that parietal cortex may process higher-order information during navigation. For example, single neurons in rat parietal cortex have been shown to reflect a “route-centered” reference frame (Nitz, 2006). These cells were activated in a similar manner across different traversals of a particular route, independent of the absolute spatial location or direction of motion. Similar neurons have been recorded from medial parietal areas in monkeys (Sato et al., 2006). These cells varied their activity across movements of the same type in the same place, but which were part of different routes. Further evidence of a non-egocentric representation of space was obtained in experiments in which lesions to area 7a in cynomolgus monkeys resulted in impairments in the traversal of whole-body mazes (Traverse and Latto, 1986; Barrow and Latto, 1996). Monkeys with these lesions had difficulty using information from visual cues to navigate. In one experiment, some monkeys relied on the locations of visual cues to navigate to the exit of the maze, while others learned a series of turns and ran the same route regardless of where in the maze they started. The area 7a lesions only affected those monkeys that used the visual cues to navigate, suggesting that this area is involved in the integration of visual landmarks in navigation.

#### Spatial representation during covert maze solution

Another realm in which spatial cognition is seen to be decoupled from stimulus and movement parameters is in the solution of visual mazes. Behavioral studies of humans and monkeys following paths in mazes suggest a covert process that analyzes the path, taking a longer time when the path is longer or has more turns in it (Crowe et al., 2000; Chafee et al., 2002). This path-tracking behavior is similar to the following of a route on a map, which is itself a spatial operation related to navigation. Imaging of human subjects who both navigated a 3-D virtual environment and viewed a top-down, or survey, view of the environment showed that many brain areas, including superior parietal cortex, were activated in both tasks (Shelton and Gabrieli, 2002).

**Figure 4:**
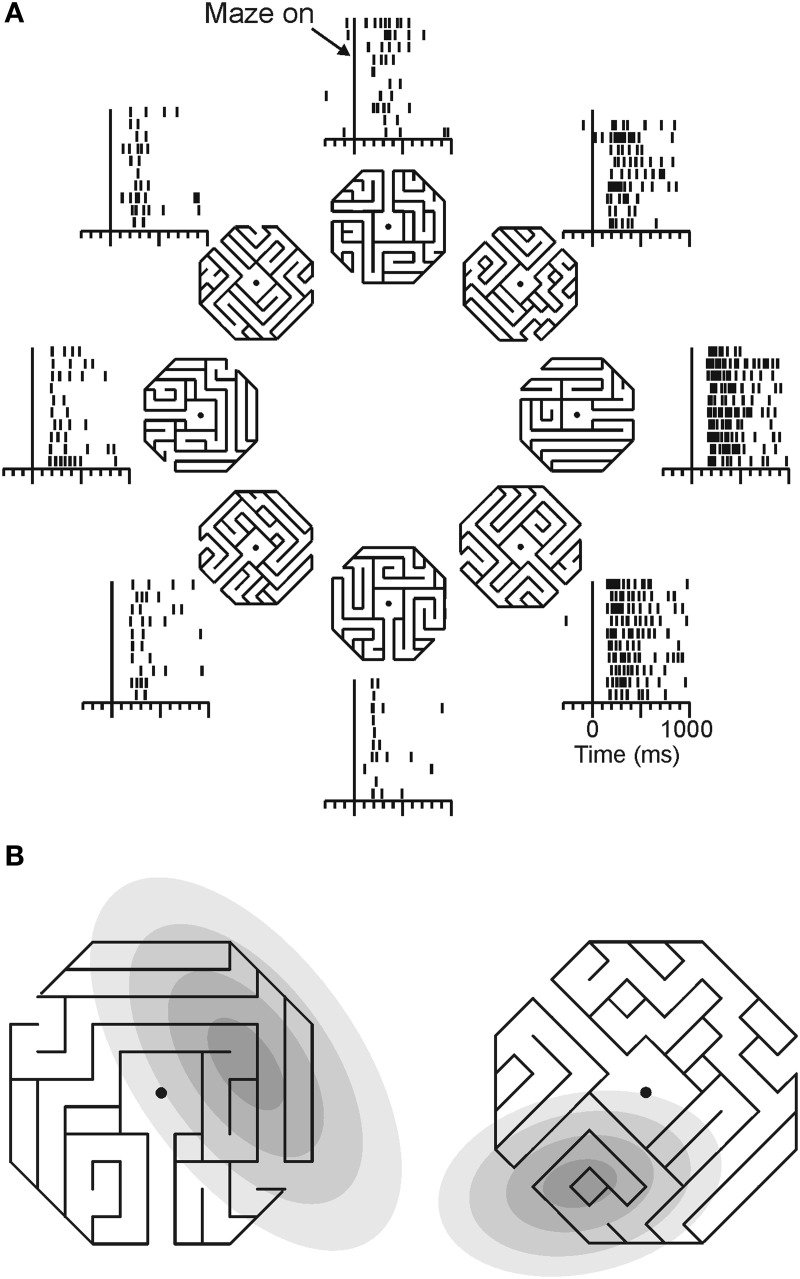
**Directional tuning of a parietal neuron during maze solution. (A)** One maze stimulus was shown on each trial (mazes were 30° of visual angle across) centered on the gaze fixation target. Monkeys were trained to indicate whether the path that emanated from the center of the maze reached an exit or ended inside the maze. Only straight exit paths are shown in this figure. Each raster shows the neural activity of an area 7a neuron during trials in which the followed path pointed in the indicated direction. **(B)** The direction of the path emanating from the center of each maze indicates the preferred maze path of each cell. Gray ellipses indicate, in the same spatial scale, the locations of each cell's visual receptive field, mapped with focal stimuli. Across the population, there was no concordance between receptive field location and preferred maze path direction.

Georgopoulos and colleagues (Crowe et al., 2004) recorded from parietal area 7a neurons as monkeys mentally followed a path within a maze displayed on a computer screen (Figure 4). During this task, about one quarter of all cells recorded showed activity that was tuned to direction of a straight path emanating from the center of the maze. An example of such a neuron is shown in Figure 4A.

This tuned activity was related to the solution of the mazes in a manner that was distinct from sensorimotor parameters. Neurons recorded from a monkey that viewed and attended to maze stimuli, but did not solve them, did not show tuning for path direction. Additionally, data from visual and oculomotor control trials showed a dissociation of neural activity during maze solution and sensory/motor processing. Of the cells that were tuned in the maze task, three quarters were *not* tuned to the direction of eye movements in a delayed saccade task, and the cells that were tuned in both tasks had tuning functions that were not systematically aligned. Maze tuning was similarly dissociated from visual stimuli in control tasks. Few maze-tuned neurons showed tuning during the cue period of the delayed saccade task, and locations of independently mapped receptive fields were unrelated to preferred maze directions (Figure 4B). Across the population of cells, there was no systematic relation between the location of the visual receptive fields and cells' preferred maze directions, suggesting single neurons could carry independent spatial signals under the two different task contexts.

**Figure 5:**
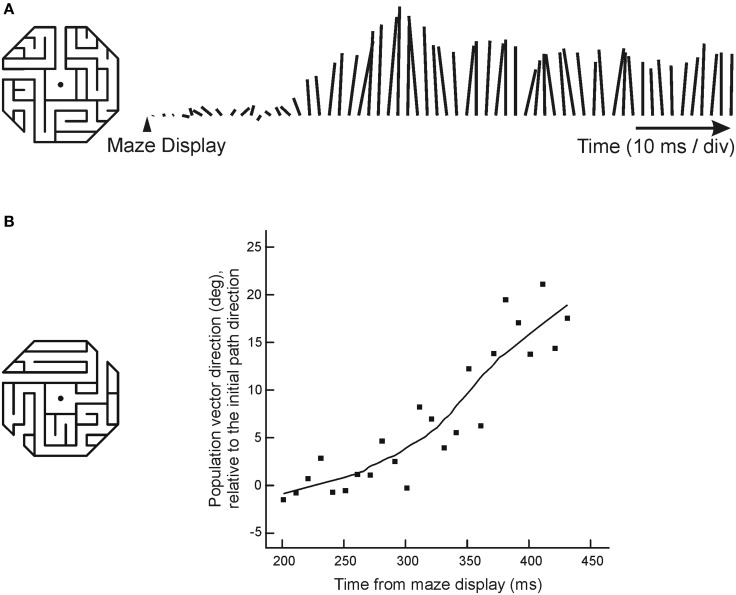
**Dynamic neural activity during maze solution. (A)** The lines to the right of the maze indicate the length and direction of the population vector, calculated every 10 ms. This example shows the behavior of the population vector during solution of mazes with straight paths that pointed up. The population vector began growing in the direction of the maze by about 200 ms, and continued to point up throughout the trial. **(B)** When monkeys solved mazes with a right-angle turn, the population vector first pointed in the initial path direction and then rotated in the direction that the path turned (positive values in the graph represent angles in the direction the path turned, negative values represent angles away from the turn).

As a final indication that this neural activity reflected a spatial cognitive process, Crowe and colleagues measured the directional tendency of the neuronal population over time during maze solution (Crowe et al., 2005). In cases when monkeys solved mazes with straight paths, the neuronal population vector (Georgopoulos et al., 1986) began pointing in the direction of the path shortly after the maze was displayed, and remained pointing in that direction over the course of the trial (Figure 5A). In trials in which the monkeys solved mazes with a single right-angle turn, the population vector rotated in the direction of the turn (Figure 5B). This change in neural activity occurred in the absence of any change in visual stimulation, and in the absence of motor output.

Interestingly, the rotation of the population vector was characterized by the subsequent activation of cells whose preferred directions pointed in the direction of the initial maze direction, and then of cells whose preferred directions pointed toward the maze exit, at an angle of 45° defined with respect to the gaze fixation target. There was no activation of cells with preferred directions 90° from the initial path direction (which would be predicted if the spatial signal in area 7a reflected the direction of movement through a path with a 90° turn). This suggested that the progression of the cognitive process following the path through the maze could be related to the neural representation of a vector with an origin that remained anchored at the fovea, and a tip that moved progressively along the maze path from origin to exit. These results, taken together, highlight the cognitive nature of these spatial signals recorded from parietal cortex, and their dissociation from sensory and motor parameters.

#### Spatial representation during object construction

Damage to the posterior parietal cortex disrupts spatial cognition, in addition to spatial attention and sensorimotor control. Constructional apraxia provides an example of a spatial cognitive disturbance seen after parietal damage that cannot be explained purely in terms of a sensory or motor deficit. Patients with this syndrome are unable to analyze and effectively reproduce the spatial structure of objects when they attempt to draw or assemble a copy of them. The copies they produce are spatially disorganized—parts are omitted and the ones included are frequently placed in the wrong positions relative to one another, so that the constructed object is disarrayed. These spatial deficits can be observed in patients that do not otherwise exhibit frank visual or motor impairments (Piercy et al., 1960; Benton and Fogel, 1962; Benton, 1967; Benson and Barton, 1970; Arena and Gainotti, 1978), suggesting a specific deficit in spatial cognition. The cognitive deficit underlying constructional impairment could reflect a reduced ability to compute task-critical spatial relationships, in that the spatial structure of an object is specified by the set of spatial relationships that locate its parts with respect to one another. As a set, these spatial relationships provide a view-invariant representation of object structure that generalizes across different object positions or orientations, and it seems likely that to facilitate operations on objects, the brain generates spatial representations of this type (Olson, 2003). Prior studies have shown that neurons in the supplementary eye fields code saccade direction in object-centered coordinates (Olson and Gettner, 1995, 1999; Olson and Tremblay, 2000; Tremblay et al., 2002; Olson, 2003; Moorman and Olson, 2007a,b). However, the existence of object-centered spatial coding in parietal cortex has been debated. A prior study examined whether LIP neurons code saccade direction in object-centered coordinates and reported largely negative results (Sabes et al., 2002). Further, although parietal lesions cause object-centered spatial neglect (Driver et al., 1994; Tipper and Behrmann, 1996), the loss of fundamentally retina-centered spatial representations could theoretically explain this deficit (Driver and Pouget, 2000).

**Figure 6:**
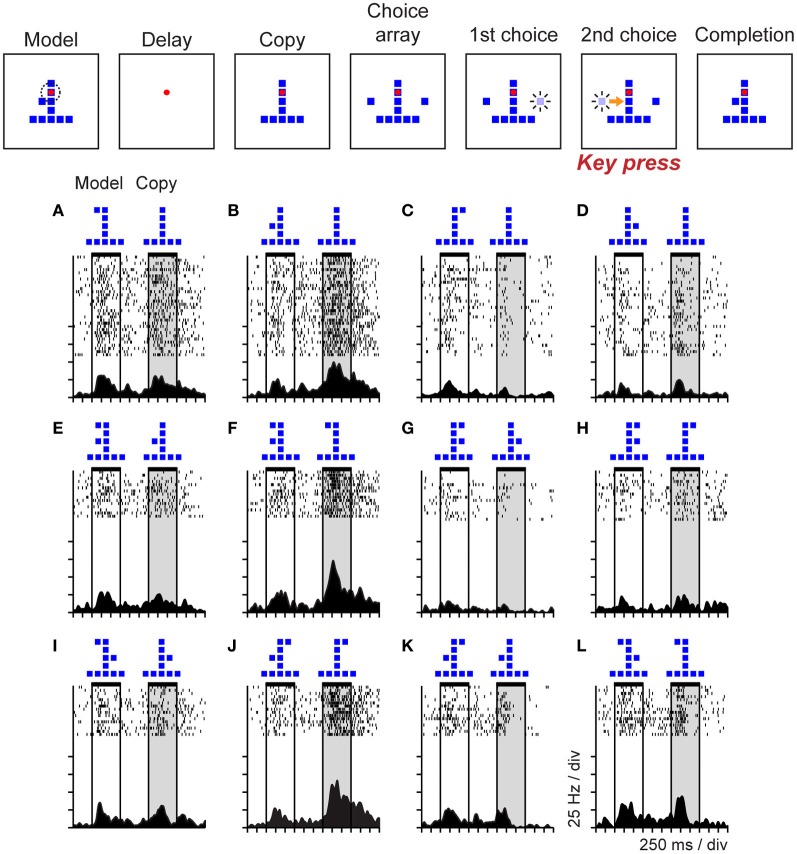
**Event sequence of the object construction task and activity of a single neuron in area 7a during task performance.** Stimuli displayed during the trial are shown in the top panel. Monkeys viewed a model followed by a copy object (each consisting of an arrangement of squares). The copy was identical to the preceding model except that a single square was missing. Monkeys had to localize the missing square and replace it to reproduce the model configuration for reward. Addition of a square to the copy was via a forced choice. Two choice squares were presented and brightened in random sequence. The monkey controlled which square was added by timing when it pressed a single response key in relation to the choice sequence (the computer added the square that was bright at the time of response to the copy object automatically). **(A–L)** The duration of model and copy periods is delimited by horizontal black bars at the top of each raster. This neuron was activated primarily during the copy period, on trials in which the model and copy objects presented jointly localized the single square missing from the copy object (relative to the preceding model) to the middle left position within the object.

To study the neural correlates of cognitive operations involved in the analysis of the spatial structure of objects, and to determine if parietal neurons might support object-centered representations of space, Georgopoulos and colleagues trained monkeys to perform an object construction task based on human clinical tests of constructional ability and recorded neural activity in posterior parietal area 7a during task performance (Chafee et al., 2005). The sequence of task events is represented at the top of Figure 6. Monkeys were presented with two objects each trial consisting of an arrangement of squares. The first object was the model that monkeys were required to copy. The second object was a partial copy of the model, identical except that one square was missing. Monkeys had to compare the structure of model and copy objects to locate the missing square in the copy object. They then replaced the missing square to reproduce the model configuration for reward. (Monkeys selected one of two sequentially presented choice squares by timing when they pressed a single response key, so movement direction did not vary over trials.)

Neural activity in area 7a varied systematically as a function of the missing square in the copy object. This provided an example of a case in which parietal activity coded a cognitive spatial variable rather than a sensorimotor one because the task was designed so that the location of the missing square did not correlate either with the retinal position of a visual stimulus or the direction of the required motor response. Rasters (Figures 6A–L) illustrate a single area 7a neuron that was activated during the copy period of the task (shaded vertical gray rectangle), but only on the subset of trials in which the configurations of the model and copy objects, taken together, jointly localized the missing square to the lower left position within the copy object. The spatial information coded by this neural activity did not derive directly from the visual features of the objects (such as position or configuration). For example, the trials illustrated across the top row (Figures 6A–D) all presented the same copy object at the same position in the visual display, but neural activity clearly varied as a function of where the monkey had determined the missing square was located on each trial. Examination of the pattern of activity across trial conditions demonstrates that activity in this neuron was not an obligatory function of the configuration of the model object shown earlier in the trial either. Nor did the spatial information carried by the activity of this neuron bear a systematic relation to the direction of motor output (which did not vary over trials). The activity of this neuron therefore appeared to reflect a process more akin to spatial problem solving, than spatial vision or sensorimotor control. The interpretation of this activity as reflecting a cognitive analysis of object structure, rather than a more basic spatial sensorimotor or attention process, is supported by the observation that this neural population was generally not activated when monkeys viewed, planned saccades, or directed attention toward visual stimuli placed at the same locations the cells preferred in the construction task (Chafee et al., 2005). The minority of neurons active during both construction and control tasks often exhibited different spatial tuning in the two contexts, a pattern we had seen during visual maze solution (Figure 4B) (Crowe et al., 2004).

**Figure 7:**
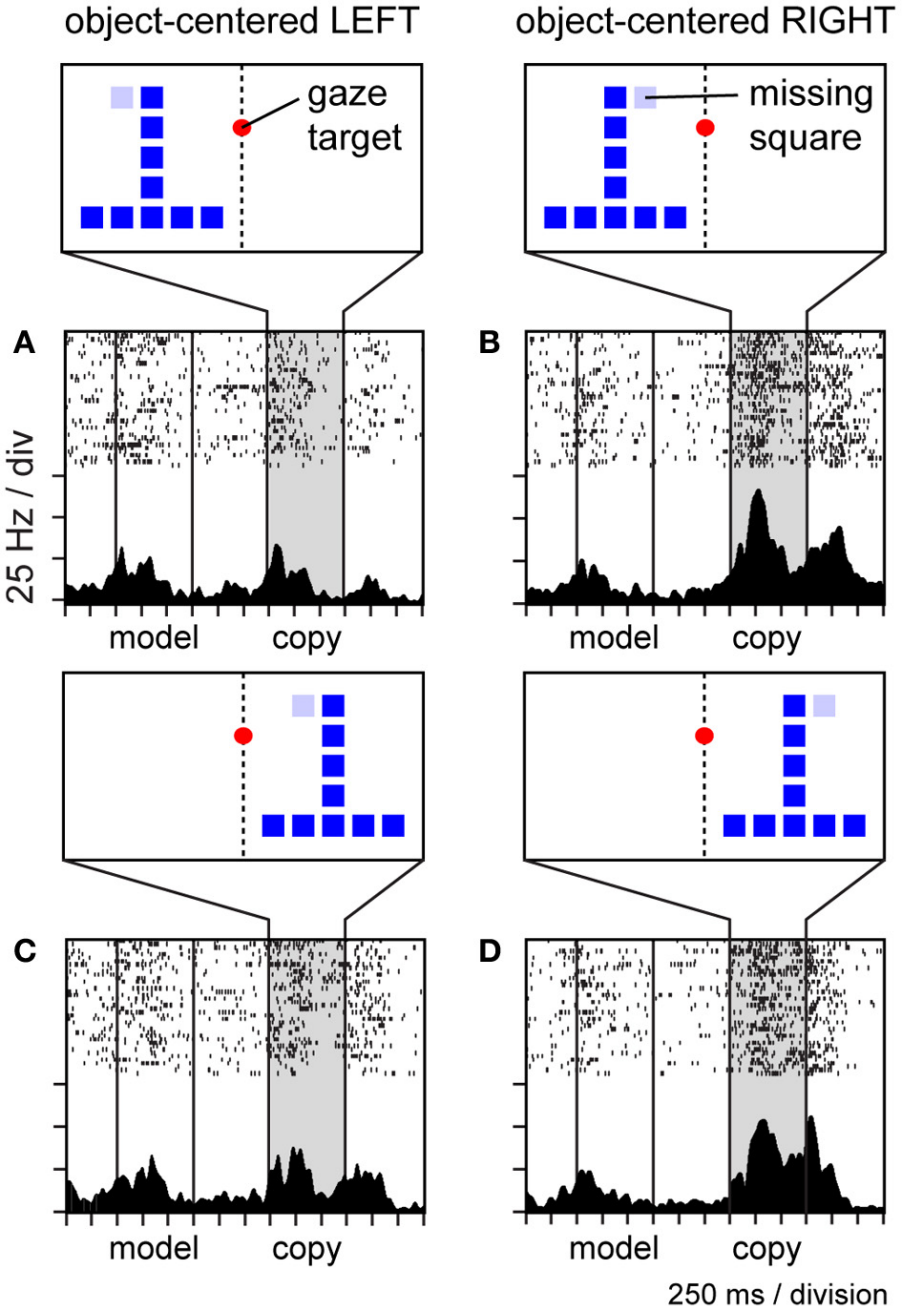
**Activity of a single area 7a neuron coding the location of the missing square in the copy object in object-centered spatial coordinates.** The location of the missing square is indicated in light blue but was not visible to the monkey (the copy object consisted only of an inverted “T” configuration of dark blue squares in these trials). The copy object was presented to the left **(A,B)** or right **(C,D)** of the gaze fixation target at random over trials. The missing square was located on the relative left **(A,C)** or right **(B,D)** side of the object, relative to its intrinsic midline, at random over trials. Consequently, the object-centered and viewer-centered side (left or right) of the missing square in the copy object were statistically independent spatial variables. This neuron was activated whenever the missing square was located on the relative right side of the copy object **(B,D)**, regardless of whether the copy object appeared in left **(B)** or right **(D)** viewer-centered space.

To explore whether neurons in area 7a might code the location of the missing square during the construction task in object-centered coordinates, we randomly shifted the position of the copy object to the left and right of the gaze fixation target (which defined the center of viewer-centered spatial frameworks), and found that a large proportion of parietal neurons were insensitive to this manipulation, coding the position of the missing square relative to the object midline in an apparently view-independent manner (Chafee et al., 2007). For example, the neuron illustrated in Figure 7 was activated when the missing square was located on the relative right (Figures 7B,D) and not the left (Figures 7A,C) side of the copy object, regardless of whether the copy object itself was presented in the left (Figures 7A,B) or right (Figures 7C,D) side of viewer-centered space. We did find that the activity of these neurons correlated with the location of covert spatial attention, as monkeys were faster to detect probe stimuli presented unpredictably at the location of the missing square in the middle of a construction trial (Chafee et al., 2007), much the same way that LIP neurons were found to signal the location of attention during the performance of a memory-guided saccade task (Bisley and Goldberg, 2003). However, the 7a neurons we studied during object construction were not generically related to spatial attention, as they failed to activate when monkeys directed attention to the same locations in different task contexts (Chafee et al., 2005). Finally, most of the neurons studied during construction preferred locations on the contralateral side of objects irrespective of the absolute locations of the objects (Chafee et al., 2007). That contralateral bias at the neural population level could potentially explain why object-centered neglect after unilateral parietal damage typically involves the contralesional side of objects (Olson, 2003).

#### Representation of spatial categories

**Figure 8:**
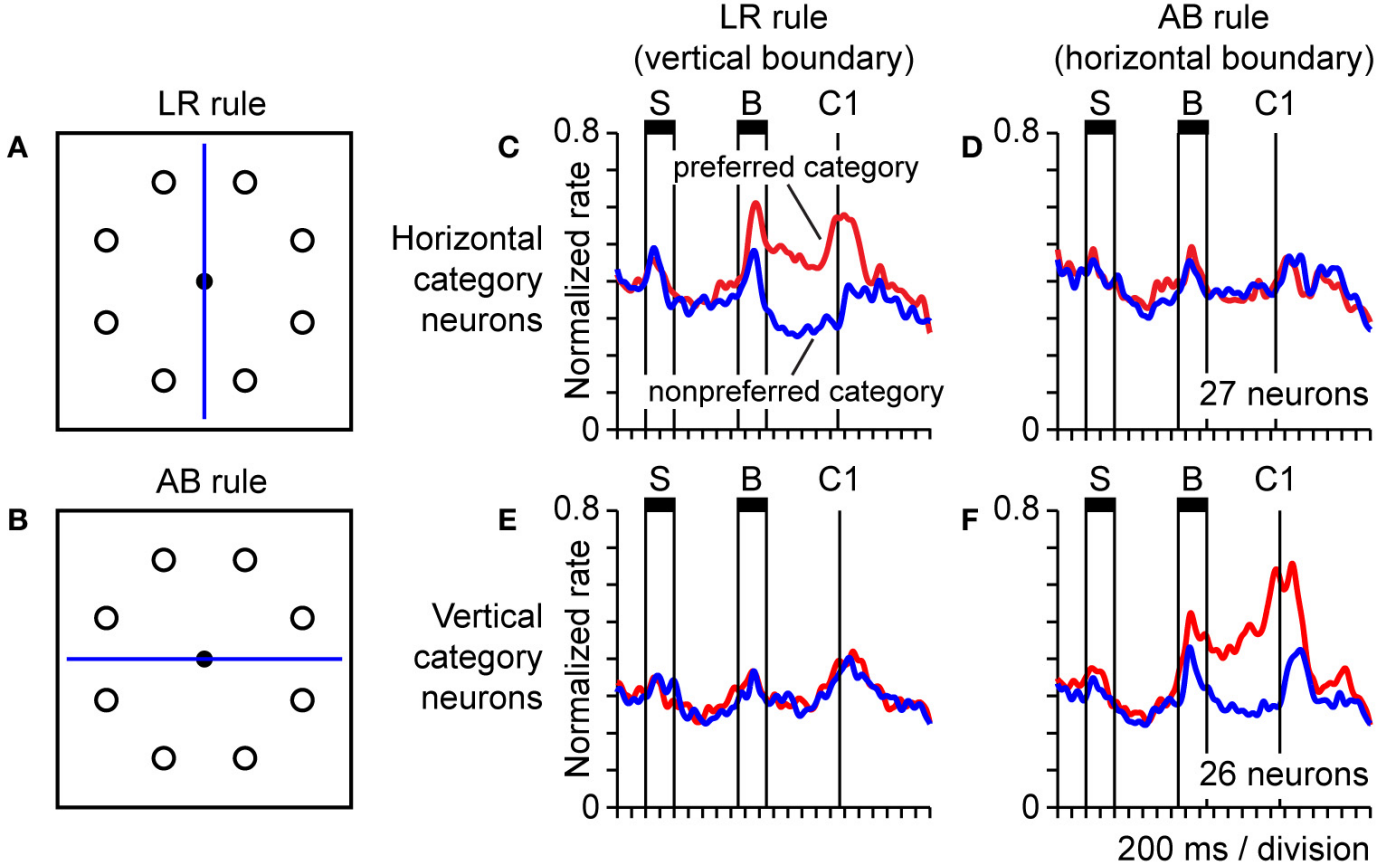
**Neural population activity encoding categorical spatial relationships in posterior parietal cortex area 7a.** Monkeys performed a task in which they assigned a spot sample stimulus to one of two spatial categories on the basis of the spatial relationship between the sample and a line serving as a category boundary. We presented the sample and category boundary stimuli at different times in the trial, separated by an intervening delay. The durations of the sample and boundary cue are indicated by horizontal bars labeled “S” and “B,” respectively **(C–F)**. Monkeys reported their categorical judgment by pressing a response key when a subsequent choice stimulus appeared in the same spatial category as the sample (the time of onset of the first choice is labeled “C1”) **(A,B)**. Circular array of sample stimulus positions and category boundary shown bisecting the array in either a vertical or horizontal orientation (the orientation of the boundary varied over trials). A vertical boundary instructed the monkey to divide the circular array of positions into the spatial categories left and right (LR rule). A horizontal boundary instructed the monkey to divide the circular array of positions into the spatial categories above and below (AB rule) **(C,D)**. Activity of a population of 27 parietal neurons coding the horizontal category of the sample under the LR rule **(C)** and not the AB rule **(D)**. Population activity is plotted separately for trials in which the sample fell in the preferred (red) and non-preferred (blue) horizontal category for each neuron, defined on the basis of the position of the sample stimulus **(E,F).** Corresponding data for a distinct population of 26 area 7a neurons coding the vertical categories above and below under the AB rule **(F)** and not the LR rule **(E)**.

Object-centered spatial codes provide an example of how neurons can carry spatial information that generalizes across a potentially infinite set of specific stimulus conditions, so long as the defining abstract feature, the spatial relationship (between a point in space and an object) holds. Spatial categories (categories defined on the basis of spatial information) are analogous in that they similarly exemplify spatial regularities or underlying principles that can be embedded in a potentially infinite set of different stimulus configurations, and prior work has shown that parietal neurons code spatial categories. For example, posterior parietal neurons code categories of visual motion direction (Freedman and Assad, 2006; Swaminathan and Freedman, 2012) and spatial position (Merchant et al., 2011) in a dichotomous fashion when these continuously varying stimulus attributes cross a learned or inferred category boundary. The finding that parietal neurons coded object-centered position (Figure 7) suggested they might also code spatial categories based on spatial relationships. To explore that possibility, monkeys were trained to place a spot visual stimulus into a spatial category on the basis of its spatial relationship to a line serving as a category boundary. Stimuli were presented in a circular array, and the category boundary bisected the array in either a vertical or horizontal orientation (Figures 8A,B), instructing either a left/right categorization rule (LR rule), or an above/below categorization rule (AB rule). This placed categorization under executive control. Population activity in parietal area 7a reflected the assignment of positions to categories in a rule-dependent manner (Goodwin et al., 2012). One population of neurons exhibited activity that dissociated left and right categories under the LR (Figure 8C) and not the AB (Figure 8D) categorization rules. Another population exhibited similar rule-dependent selectivity for vertical categories (Figures 8E,F). Activity of this type was dissociated both from the position of the stimulus and the orientation of the boundary, as the rule-dependent category information coded by cells was jointly defined by both factors taken together, and therefore dissociated from either considered individually.

### Origin of third order spatial representations

One of the most important questions regarding the hierarchy of spatial representation found in parietal cortex is how neural signals coding abstract spatial information (such as spatial categories or relative positions) derive from simpler sensorimotor signals. The answer should provide insight, perhaps of general scope, into how neural systems acquire the capacity for abstraction as a function of sensorimotor experience. In the practical context of most neurophysiological experiments, this amounts to understanding how abstract neural signals in the brain emerge as a function of training to perform a particular behavioral paradigm. Cognitive paradigms developed for monkeys are generally of a relatively simple form; however they capture something fundamental to more elaborate forms of cognition in humans. The brain has to generate a set of cognitive representations that capture an implicit principle of general applicability embedded in a set of superficially disparate stimuli or events. Neural signals coding spatial categories provide one concrete example (Figure 8). The grouping criteria governing category membership, based on a spatial relationship in this case, is the generalized principle, which could be applied to categorize a potentially infinite set of exemplars. Once these abstract representations emerge, the brain then has to discover the correct mappings between sensory input, cognitive signals, and motor commands.

We know comparatively little about the neural mechanisms by which repeated experience with the world leads to the emergence of cognitive neural signals in the cortex, and the capacity for abstraction and prediction in novel circumstances that these signals are likely to underlie. However, there is little doubt that reward processing and reward-modulated synaptic plasticity play an essential role, although the role of training may differ for second and third order processes as defined above. For example, there is interesting evidence that signals that reflect working memory (a second order process) in prefrontal cortex are present in experimentally naïve animals before training (Meyer et al., 2007), although the information encoded by this activity increases with training (Meyers et al., 2012). That suggests that there exists, to a certain degree, a native working memory capability continually operating in the background to effectively buffer the sensory input regardless of its learned behavioral significance. However, it seems equally like that most of the third order cognitive signals described, such as those coding abstract categories of experimental stimuli, or rules governing task contingencies, did not pre-exist in the cortex of monkeys prior to training. In fact there is evidence that training exerts a powerful effect on category-selective neural signals both in parietal (Freedman and Assad, 2006) and prefrontal (Freedman et al., 2001; Cromer et al., 2010; Roy et al., 2010) cortex. To our view, the fact that most third order spatial cognitive signals so far described are likely to be “trained into” the brain does not undercut the utility of this general experimental approach for studying the neural origins of abstraction at the single cell level. We would argue that similar processes are taking place in the human brain continuously, given that it is likely we learn much of our abstract knowledge by interacting with a statistically structured environment (Tenenbaum et al., 2011), coupled with reward history. Behavioral paradigms used to study neural correlates of cognition in monkeys are formalizations of these same features. To enable abstraction, neurons at higher levels of the cortical processing hierarchy have to detect and extract statistical regularities (perhaps relating to generalized principles or “knowledge”) embedded in activity at lower levels, a process that can be effectively modeled in the non-human primate. We know that this process takes place, but how is one of the most important unanswered question presently confronting cognitive neuroscience.

The integration of statistical models of human cognition (Tenenbaum et al., 2011), with theory-informed biological experiments is likely to lead discovery of the neural mechanisms that generate a capacity for abstraction in neural systems. From that perspective, biological data that can test predictions based on theory will be particularly important. Many models of human cognitive processes can be probed to make predictions about how information should flow between populations of neurons that encode different types of behavioral or cognitive information, as sensory inputs are transformed into more abstract cognitive signals to control behavior, for example. We sought evidence of this type of communication between simultaneously active neural populations coding different types of information in posterior parietal cortex. More specifically, we measured short-term fluctuations in the amount of information about a spatial location coded by two different populations of neurons that were coactive in parietal cortex during the object construction task. The first population coded the position of object squares in a retinocentric, or viewer-centered framework, and therefore provided an example of a first order spatial representation. The second population coded the position of object squares in object-centered coordinates. Because object-centered positions are intrinsically relational (and abstracted from specific absolute positions), signals coding them constitute an example of a third order spatial process. To measure interactions between groups of neurons coding these two types of spatial information, we first measured the firing rates of ensembles of neurons coding position in viewer-centered and object-centered coordinates that we had recorded simultaneously, and applied a pattern classification analysis to these firing rates to quantify short-term fluctuations in the strength of the signals coding space in the two coordinate frames. We then employed Granger causality analysis to examine the temporal correlation between the two time series (after accounting for their autocorrelation). Using this approach it was possible to determine that fluctuations in the strength of the viewer-centered signal preceded and predicted variation in the object-centered signal, but not the converse, and only in the case that the groups of neurons representing the two types of spatial information were recorded simultaneously (Crowe et al., 2008). That provided physiological evidence that abstract neural representations at higher levels of the cortical processing hierarchy receive input and derive from signals at lower levels. We also found that viewer- and object-centered representations of space exhibited markedly different population dynamics. Viewer-centered position was represented by a pattern of population activity that was relatively stable over time, whereas object-centered position was represented by a pattern of population activity that was continuously evolving and highly dynamic, such that subsets of cells carrying the same spatial information were briefly activated in rapid and repeatable sequence throughout the trial (Crowe et al., 2010). These data suggest that distinct neural mechanisms are employed to represent spatial information at different levels of the hierarchy in parietal cortex.

It is important to note that parietal neurons encode non-spatial cognitive variables as well, and non-spatial information can coexist with spatial sensorimotor information in the activity of single neurons (Gottlieb and Snyder, 2010). For example, individual LIP neurons can encode both task rules and movement direction (Stoet and Snyder, 2004), or can carry signals that reflect task context and stimulus position (Balan and Gottlieb, 2006), often at different times in a single trial. This combination of signals in LIP neurons may bias the neural representation of space to reflect cognitive factors or represent an intermediate step toward the generation of purely cognitive signals. However, interestingly, inactivation of LIP neurons appears to impair behavior primarily by interfering with spatial selection, leaving the ability to modulate behavior according to non-spatial cognitive factors relatively intact (Balan and Gottlieb, 2009). That suggests that neural signals in LIP encoding non-spatial cognitive factors may reflect top-down input from other cortical structures.

The data reviewed above provides evidence that neural representations of space that exhibit sensorimotor independence in posterior parietal cortex (1) are mediated by context-sensitive signals distinct from those coding stimulus or motor parameters, (2) still bear a relation to spatial attention (but not in a way dictated directly by sensory input), (3) may emerge by virtue of a transformation applied to population activity coding stimuli and movements, and (4) appear to be mediated by population activity that exhibits unique temporal dynamics.

## Summary and conclusion

Perhaps the single most fundamental fact to emerge from the experimental evidence reviewed above is that posterior parietal cortex sustains a hierarchy of spatial representations, which exhibit different relations to behavior and appear to be mediated by distinct neural mechanisms. One of the key dimensions differentiating the levels of this representational hierarchy is sensorimotor independence, the degree to which spatial information coded by parietal neurons remains tightly coupled to stimulus and motor parameters, vs. the degree to which spatial representations diverge from sensorimotor factors to mediate various forms of spatial reasoning or problem solving that could be considered to constitute instances of spatial intelligence. The long-standing debate as to whether spatial signals carried by parietal neurons reflect stimuli vs. movements, or visual attention vs. motor intention, has produced compelling evidence in favor of both conclusions, suggesting that these are not mutually exclusive. Even in the more abstract case that parietal neurons represent spatial locations dictated entirely by cognitive rather than sensorimotor factors, neurons appear to continue to reflect the location of spatial attention. From that perspective, biases in both sensory and motor processing could be considered simultaneous corollaries of spatial information represented in parietal cortex. A critical question remaining is how abstract spatial representations in parietal cortex are learned, or more specifically, what are the neural mechanisms that derive them from lower level spatial sensory and motor representations in this area. That question seems experimentally approachable, and integration of experimental and theoretical work stands to provide substantial insight into the neural mechanisms involved.

### Conflict of interest statement

The authors declare that the research was conducted in the absence of any commercial or financial relationships that could be construed as a potential conflict of interest.
